# 2020 *Micromachines* Yong Investigator Award: Announcement and Interview with the Winner

**DOI:** 10.3390/mi12010048

**Published:** 2021-01-02

**Authors:** Nam-Trung Nguyen

**Affiliations:** Queensland Micro- and Nanotechnology Centre, Griffith University, 170 Kessels Road, Nathan, Queensland 4111, Australia; nam-trung.nguyen@griffith.edu.au

After an extensive voting period, we are proud to present the winner of the *Micromachines* Young Investigator Award to:


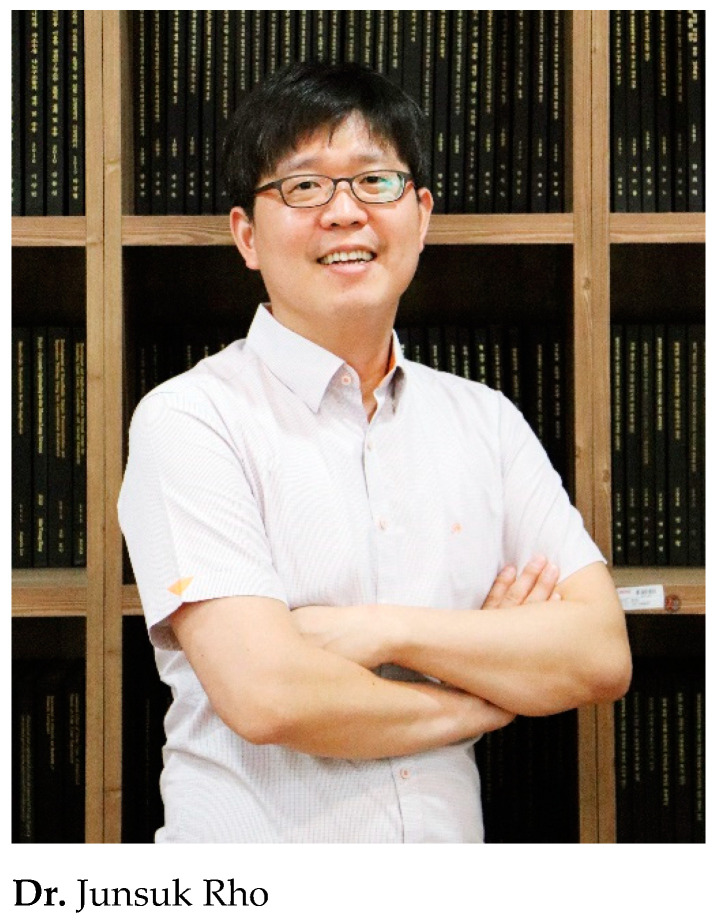


Junsuk Rho is currently a Mu-Eun-Jae endowed Chair Associate Professor with a joint appointment in the Department of Mechanical Engineering and the Department of Chemical Engineering at Pohang University of Science and Technology (POSTECH), Korea. Before joining POSTECH, he received his B.S. (2007) and M.S. (2008) degrees in Mechanical Engineering at Seoul National University, Korea, and the University of Illinois, Urbana-Champaign, respectively. After receiving his Ph.D. (2013) in Mechanical Engineering and Nanoscale Science and Engineering from the University of California, Berkeley, Junsuk Rho worked as a postdoctoral fellow in the Materials Sciences Division at Lawrence Berkeley National Laboratory and Ugo Fano Fellow in the Nanoscience and Technology Division at Argonne National Laboratory. His research is focused on developing novel nanophotonic materials and devices based on fundamental physics and experimental studies of deep sub-wavelength light–matter interaction. Dr. Rho has published approximately 150 high-impact peer-reviewed journal papers and has also presented keynote and invited talks more than 250 times at world-leading institutes and international conferences/workshops. He also has 4 US patents and 26 Korean patents.

On behalf of the *Micromachines* Editorial Office staff and award evaluation committee, I congratulate Dr. Junsuk Rho on his excellent performance and wish him all the best for his future career.

## Interview with the Winner

### 1. Could you Give us a Brief Introduction of Yourself to the Readers?

This is Junsuk Rho, who is currently a Mu-Eun-Jae endowed chair Associate Professor with a joint appointment in the Department of Mechanical Engineering and the Department of Chemical Engineering at Pohang University of Science and Technology (POSTECH), Korea.

### 2. What’s your Current Research and why did You Choose this Research Field? 

My research is focused on developing novel nanophotonic materials and devices based on fundamental physics and experimental studies of deep sub-wavelength light-matter interaction. Specifically, I am working on metamaterials, plasmonics, photonic crystals, topological photonics. Recently, the wavelength regime has expanded to longer wavelengths such as acoustic and elastic frequencies. I saw the news that invisibility cloak may be realized by metamaterials. That's the reason why I want to do this for my Ph.D. study. Such metamaterials will be able to realize invisibility cloaks, super-resolution imaging, acoustic black hole, hologram display and so on.

### 3. Which Research Topics do you Think are of Particular Interest to the Research Community in the Coming Years?

Quantum Optics, Flexible Opto-Electronics, Photonic Chips for diagnostics, Single-digit-nanometer fabrication to overcome the current semiconductor industry.

### 4. Have You ever Encountered any Difficulties when You Conduct Research? How did You Overcome Them?

Everyday... This is related to #5. Have patience, try the research until it comes through without so much depressed and frustrated.

### 5. What Qualities do you Think Young Scientists Need?

Patience, Positive mind for failure (e.g. not depressed/frustrated when the experiments failed, but try the same thing in the next day!), Catching up the trends. 

### 6. Micromachines is an Open Access Journal. How do you Think Open Access Impacts the Authors?

Open access journals allow more readers to read the papers, which is very good to share the result with the communities. However, with the limited funding (especially for junior researchers), it sometimes becomes a burden. So, some kinds of supports for junior researchers are necessary. In terms of this, MDPI does a very good job to provide financial support and waive of the fee for the junior researchers.

